# Neural Induction from ES Cells Portrays Default Commitment but Instructive Maturation

**DOI:** 10.1371/journal.pone.0001349

**Published:** 2007-12-19

**Authors:** Nibedita Lenka, Saravana Kumar Ramasamy

**Affiliations:** National Centre for Cell Science, Ganeshkhind, Pune, Maharashtra, India; University of Washington, United States of America

## Abstract

The neural induction has remained a debatable issue pertaining to whether it is a mere default process or it involves precise instructive cues. We have chosen the embryonic stem (ES) cell model to address this issue. In a devised monoculture strategy, the cell–cell interaction availed through optimum cell plating density could define the niche for the attainment of efficient *in vitro* neurogenesis from the ES cells. The medium plating density was found ideal in generating optimum number of progenitors and also yielded about 80% mature neurons in a serum free culture set up barring any exogenous inducers. We could also demarcate and quantify the neural stem cells/progenitors among the heterogeneous cell population of differentiating ES cells using nestin intron II driven EGFP expression as a tool. The one week post-plating was determined to be the critical time window for optimum neural progenitor generation from ES cells that helped us further in purifying these cells and in demonstrating their proliferation and multipotent differentiation potential. Seeding cells at varying densities, we could decipher an interesting paradoxical scenario that interlinked both commitment and maturation with the initial plating density having a vital influence on neuronal maturation but not specification and the secretory factors were apparently playing a key role during this process. Thus it was comprehended that, the neural specification was a default process independent of exogenous factors and cellular interaction. Conversely, a defined number of cells at the specification stage itself seemed critical to provide an auto-/paracrine means of signaling threshold for the maturation process to materialize.

## Introduction

Neurogenesis is a complex cyto-architectural enigma that involves intricate organizational events during embryogenesis and requires precision to the minutest extent. In fact, it involves an intelligent interplay of different developmental factors regulated intrinsically by the developing embryo and/or by extrinsic influencing factors. The paradox being whether the neurogenesis represents a default state [1], [2] or it involves a more authentic signaling cascade during early embryonic development, remains still unresolved. A recent report [3] using embryonic stem (ES) cells while does support the latter phenomenon on neural differentiation, van der Kooy's group [4] interprets it otherwise. Never-the-less, the convergence of temporo-spatial regulation and expression of several cytokines, growth factors etc. and their interaction milieu lead to the setting up of a fate decision machinery in ES cell system distinguishing the self-renewal state from lineage commitment and specification. The contribution of LIF is well documented as a key cytokine in the maintenance of undifferentiated state in murine ES cells [5]–[7] with its withdrawal triggering differentiation. In fact, the BMP or the BMP induced Id protein in concert with LIF modulates the cell fate decision machinery opting between self-renewal and differentiation [7]. However, LIF is not mandatory for human ES cells maintenance [8], [9]. There are also reports [10]–[12] indicating the existence of LIF independent pathways for ES cells maintenance where “nanog” plays an important role. Additionally, LIF also plays a critical role in the generation of an intervening primitive neural stem cell stage during neural commitment of unspecialized ES cells [4]. Hence, the ES cells maintenance and the differentiation need to be tightly regulated opting between the permissive and instructive mode of differentiation and the threshold of specific molecular determinants might modulate the balance between the two.

Cell–cell communication is an important phenomenon both during development and differentiation. In fact, cell aggregation promotes the intercellular communication during the differentiation of ES cells and thereby helps transducing the signals and sets up the milieu for lineage commitment and differentiation. The fine tuning of these event cascades is brought about by both cell intrinsic and extrinsic cues. Studies till date have focused on various factors [13], [14] to implicate their influence on overall neurogenesis with the read out emphasizing neuronal differentiation in particular. In fact, these factors could actually influence neural induction either by increasing the mitotically active progenitor pool or in helping the post-mitotic differentiation. The concrete understanding of the same would require a better model system. Accordingly, here we have used the ESC system to track independently the neural induction; differentiating the progenitor stage from the maturation state for assessing the importance of underlying cues influencing neurogenesis in a stage/development specific manner. The commonly followed strategy for neural differentiation involves the generation of three dimensional embryoid bodies (EB) from ES cells [15]–[17]. However, Smith's group [3] has demonstrated a monolayer culture without intervening EBs for neural differentiation in a medium supplemented with B27. Similarly, vitamin B12 and heparin could promote neuronal differentiation from ES cells in a serum free medium [18]. Our investigation however, has outlined the critical influence of optimum cell density in fostering cell–cell interaction in a serum free monoculture that has helped defining the niche for efficient neurogenesis from ES cells *in vitro* without any exogenous inducers.

We have carried out the neural induction in a 2D culture set up to understand its default state. Cells were differentiated successfully into neural population even in the absence of any extrinsic factors. In fact, we could demarcate the neural progenitors using the nestin enhancer mediated cell trapping approach and could quantify the same that remained identical irrespective of the plating density. However, the differentiation efficiency depended on the initial cell seeding density. We could generate about 80% neurons with a better efficiency seen at medium plating density compared to that at very low or high ones. Interestingly, the transcripts levels of certain secretory factors especially Wnt corresponded well with the cell density thus implying that, the neural induction was influenced by the concentration/dilution of factors not from the medium but through the auto-/paracrine effect of the cells themselves. Our study in fact, revealed the importance of the initial seeding density that had a direct bearing and more pronounced effect on the maturation than the initiation stage of neurogenesis; thus hinting at the maturation and hence the overall neural induction process to be an instructive event, while the commitment alone represented the default state.

## Results

### Monolayer vs. three dimensional cultures

**Figure 1:**
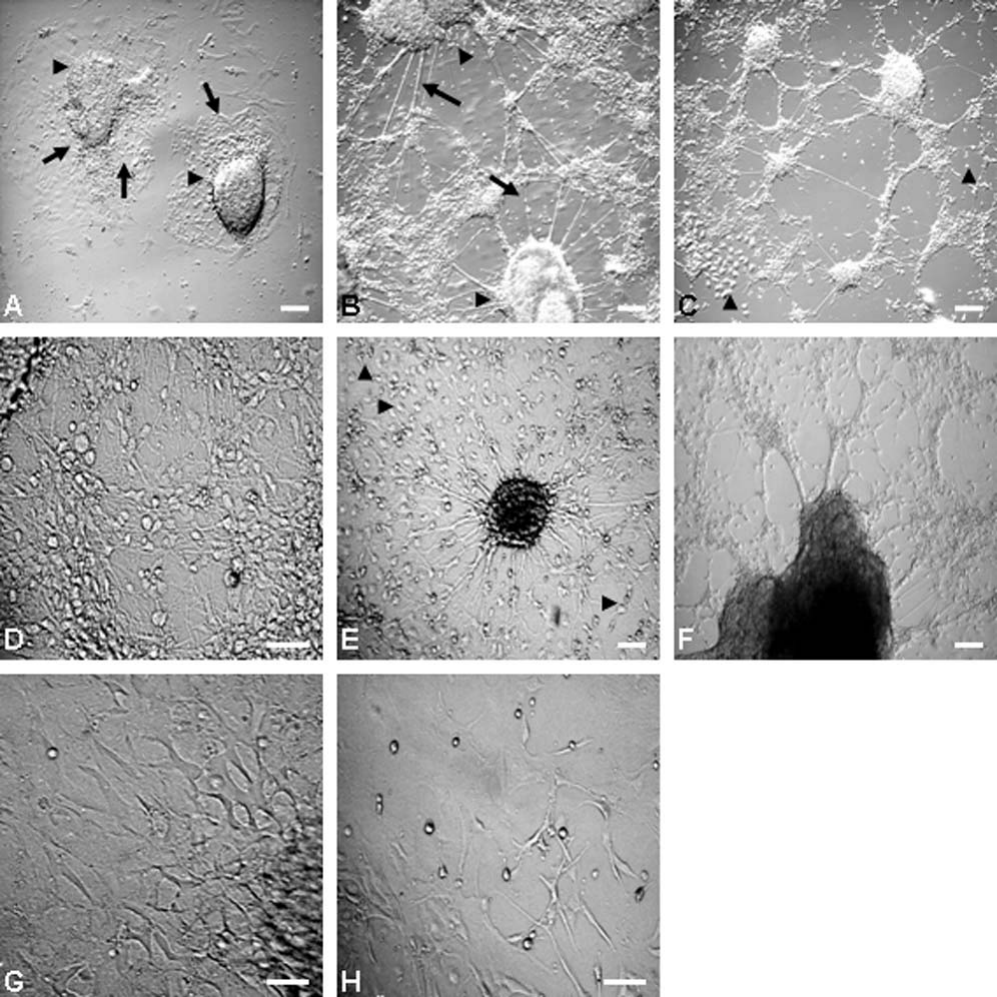
The neural differentiation was more pronounced in KO medium in both monolayer and EB cultures. The ES cells line, D3 when grown in a monolayer culture in serum containing medium for 2 days followed by 6 days (8 dpp) in KO (A) or KR (B) medium displayed EB like morphology with cell migration in a centrifugal fashion and processes sprouting (arrow) from the cell dense central part (arrowhead). RA expedited the neural differentiation process that was often associated with extensive interconnections and lattice formation. (B, C). The differentiation was comparable in KO medium at 13 dpp with (E) or without (D) RA exposure. The EBs generated by hanging drop and exposed to RA also exhibited extensive neural differentiation (d7+7) upon plating when cultured in KO medium (F). The ES cells displayed a cellular mat (G) when cultured in DM in a monolayer without RA exposure (13 dpp), while in presence of RA (H) had cells with neural morphology. Scale: 30 µM.

An adherent monolayer culture strategy was devised for neural induction from ES cells using D3 and h-nestin EGFP transfected ES cell clones (nes-EGFP). The single dispersed cells when cultured in differentiation medium (DM), generated colonies in a two-dimensional fashion due to their self renewal characteristic. On day 2 (d2), the serum containing medium was substituted with serum replacement medium (KO) and this resulted in initial proliferation and a gradual switching to differentiation. The supplementation of RA (KR) expedited this process further. The colonies in fact, grew in diameter and formed cell clusters often resembling the plated EBs (Fig. 1A, B; arrowhead). A number of smaller processes were seen sprouting from the periphery of these clusters along with the migration of single cells (Fig. 1A, B; arrows). In fact, many such clusters established interconnections forming lattices (Fig. 1B, C) during differentiation (d10 onwards). While the differentiation pattern with proper neurite extensions was apparent in KR medium by 7–8 days post-plating (dpp) (d2+5/6), a comparable status was achieved in KO medium 1–2 days later (Fig. 1B–E). However, by the second week of plating (12 dpp or later) an identical differentiation pattern was observed in both KO and KR (Fig. 1D, E). Interestingly, the KO medium also supported extensive neural differentiation in EBs in 3D culture [17] by 7 dpp (d7+7) (Fig. 1F).

### Influence of Medium and plating density on neuronal precursor generation and differentiation

**Figure 2:**
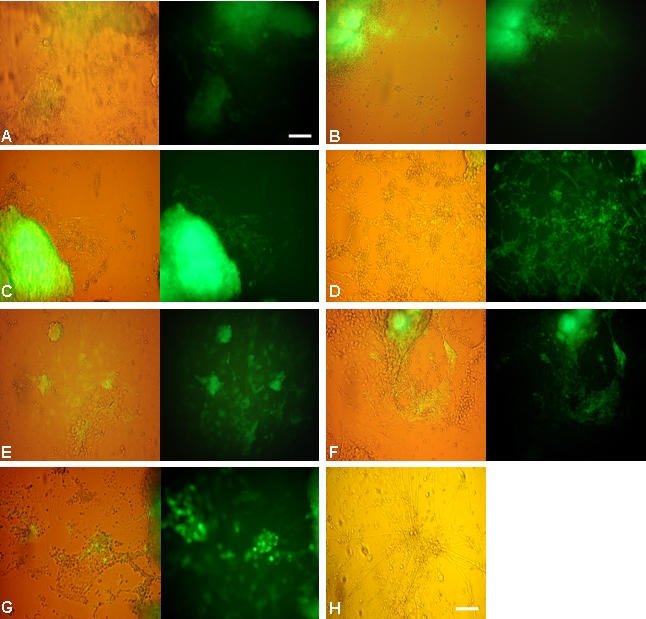
The influence of medium on neural differentiation profile in nestin transgenic ES cell clones. The differentiation pattern in nes-EGFP cells as seen in bright field and fluorescence at 8 dpp and 10 dpp in KO (A,B and C,D) and FBS containing (E,F and G) medium respectively. The neural differentiation was promoted in KO medium (A–D) compared to that in serum containing one (E–G). RA expedited the process with better differentiation in respective media (B, D and G). While the neural progenitors were EGFP^+^, the mature ones with extensive processes were EGFP^−^ (H) corresponding with the endogenous nestin expression pattern. Scale: 30 µM.

**Figure 3:**
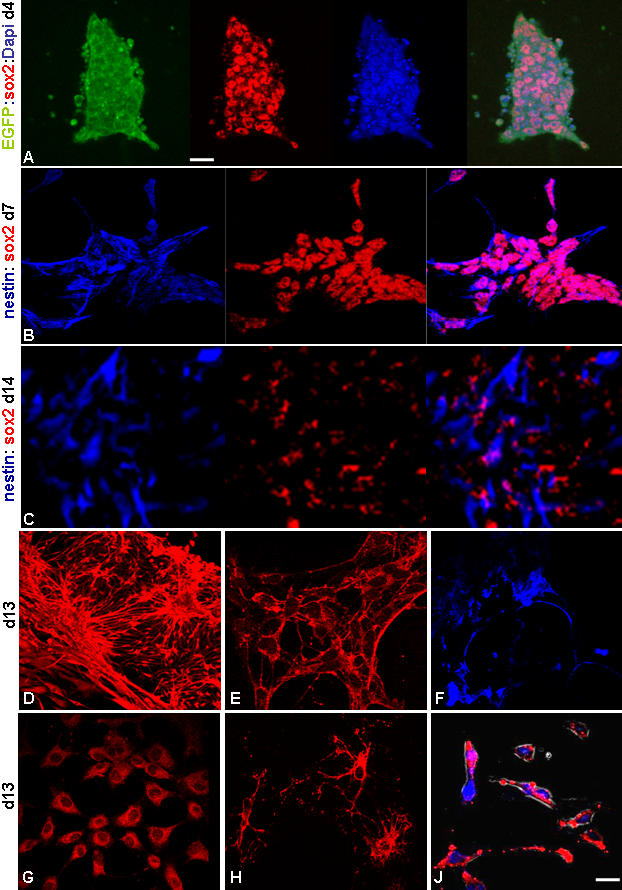
The neural differentiation and immuno-cytochemical characterization. The expression of early neural markers Sox2 and nestin was detected in an overlapping fashion at various time periods during differentiation (A–C) with Sox2 showing nuclear localization (Dapi^+^: A). Extensive neuronal differentiation was demonstrated by Map2 stained cells (D) including 5-HT^+^ serotonergic (E) and TH^+^ dopaminergic (F) neuronal subtypes. This 2D culture strategy also yielded GFAP^+^ astrocytes (G) and O4^+^ oligodendrocytes (H). The Map2^+^ cells were also synaptophysin+ (J; red: synaptophysin, blue: Dapi). Scale: 40 M except D and G (10 µM).

Our earlier study [17] had indicated an asynchrony in neural differentiation from ES cells in a 3D culture. To discern the pattern in 2D set up and optimize the same, we tested the efficacy of various media using D3 and nes-EGFP cells. The cells grown in DM formed a cellular mat with indiscernible neural structures (Fig. 1G; Fig. 2E), while with RA exposure the neural differentiation was promoted (Fig. 1H; Fig. 2F, G) indicating the inductive influence of RA on neurogenesis. The ITSFn medium with or without RA did not support the long term 2D culture, that instead showed pronounced cell death (data not presented). Among the media tested both KO and KR supported the neural differentiation most effectively (Fig. 1). In fact, by the second week of differentiation (d2+11) the entire dish was filled with Map2^+^ intricate neuronal mosaics (∼80%) (Fig. 3D). A similar profile was also observed in case of the nes-EGFP clone that upon differentiation could generate EGFP^+^ early differentiating neural cells (Fig. 2A–G) and EGFP^−^ mature ones (Fig. 2H). As seen in Fig. 2, the neurogenesis was more pronounced in KR medium (Fig. 2B, D, H) followed by that in KO (Fig. 2C).

**Figure 4:**
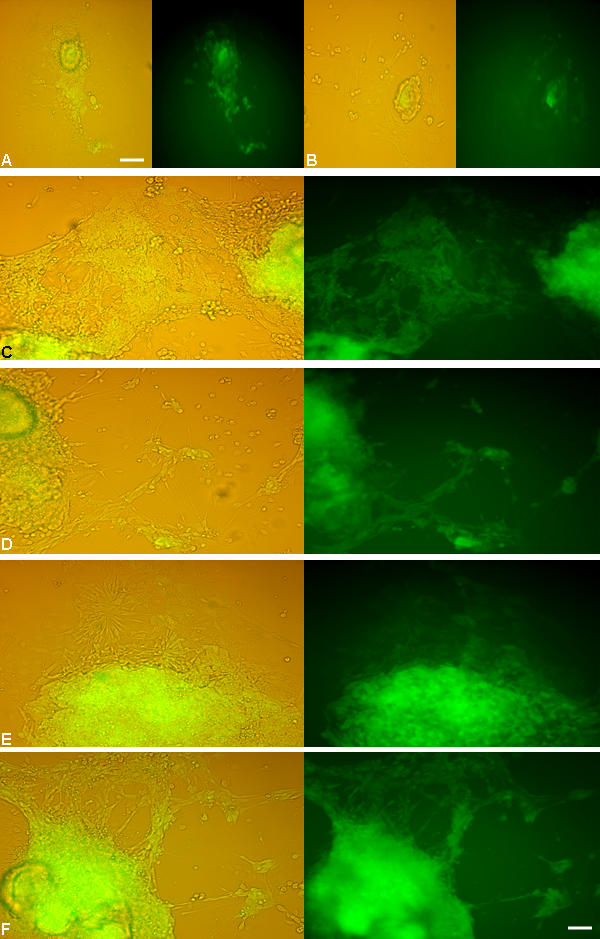
The influence of plating density on neural progenitor generation and differentiation in nes-EGFP ES cells. The cells plated at 2 K density showed EGFP^+^ neural progenitors in KO (A) or KR (B) medium, however, without any apparent differentiating neural cells at 8 dpp. The cells plated at medium (60 K; C, D) and high (130 K; E, F) densities showed prominent neural differentiation at the said time period. RA exposure (D) and (F) at the respective densities facilitated pronounced neural sprouting. Figures are shown in bright field and fluorescence. Scale: 30 µM.

Interestingly, migratory single cells in many areas in the periphery of the clusters also exhibited smaller processes extending from the cell body (Fig. 1C, E; arrowhead and Fig. S1G, H). This indicated probably the single cells also possessed independently the neurogenic differentiation potential and simultaneously questioning whether it was a density dependent phenomenon or was independent of the cell plating density. To address this issue, the cells were seeded at varying densities and the medium was changed to KO/KR on day 2 of plating. The cells at low density (2 K/35 mm) plating remained either single or formed 2–6 celled clusters on d2 of plating exhibiting slower growth (Fig. S1A) compared to the medium and high density plated ones (Fig. S1B, C). Moreover, the cells in the low density cultures lacked apparent neural differentiation (Fig. 4A, B), that was seen occasionally on the second week after plating and mainly in cell dense areas (Fig. S1D, E). Conversely, the cells when present singly or were sparse exhibited flattened morphology (unpublished data). Additionally, no differentiation was obtained with serial dilution and single cell plating in a 24- multi-well dish where single cell plating did pose a survival challenge (unpublished data). However, the cells in medium (50–65 K) or high (100–130 K) density plated cultures on 35 mm dish underwent proper neural differentiation with well sprouted neurite processes (Fig. 4C–F; Fig. S1F–H). The density higher than 100 K at times led to pronounced cell death because of cell overgrowth and hence required frequent medium replenishment. Overall we could decipher a combinatorial effect of medium plating density and KO medium on efficient neurogenesis using both D3 and nes-EGFP ES cells and RA exposure facilitated in expediting this process (compare Fig. 1A and B; Fig. 2A and B).

### Differentiation Profile

**Figure 5:**
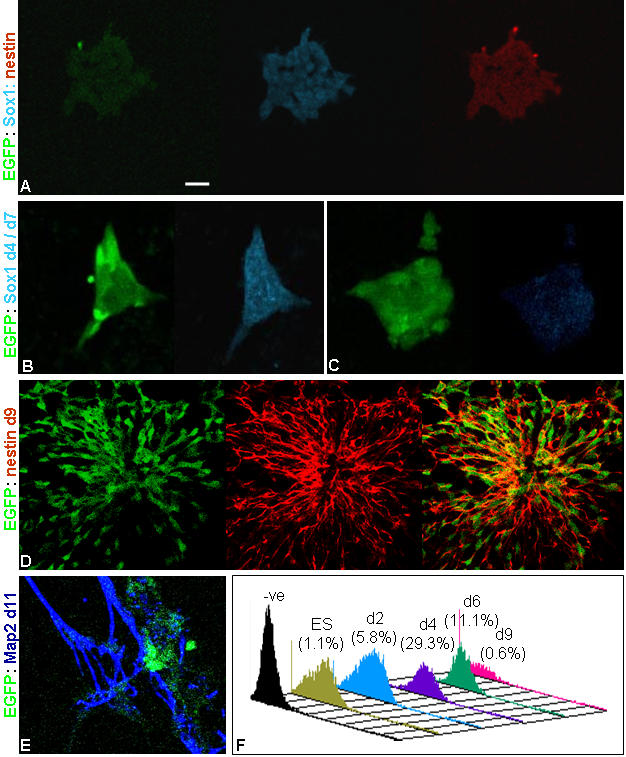
Immunophenotyping of nes-EGFP cells during differentiation. The nes-EGFP cells expressing EGFP during differentiation also expressed nestin and Sox1 as seen at 2 dpp (A). Contrary to nestin, the Sox1 expression window was transient with enhanced expression at 4 dpp (B) that became negligible by 7 dpp (C). The nes-EGFP cells exhibited rosette like structures during differentiation with co-localization of EGFP and endogenous nestin (D). The Map2^+^ differentiating neurons were EGFP^−^, however remained associated with EGFP^+^ neural progenitors (E). The histogram represents the Sox1 population during differentiation as quantified by flowcytometry (F). Scale: 40 µM.

**Figure 6:**
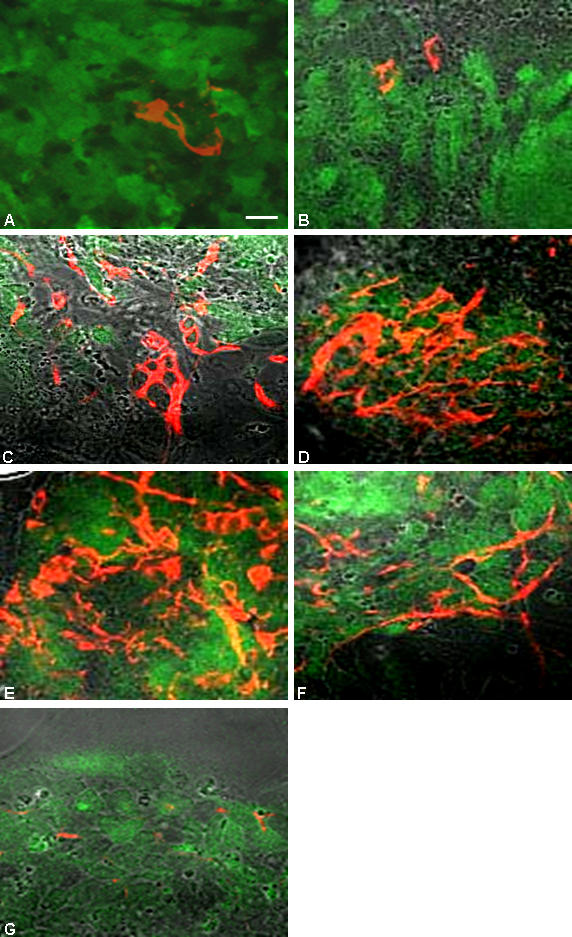
The temporal profile of neuronal differentiation in nes-EGFP cells. A few Map2^+^ neurons were detected as early as 6 dpp in cells seeded at medium density and cultured in KO (A) or KR (B) medium. The Map2 expression was extensive at 9 dpp in cells cultured in either KO (C, E) or KR (D, F) medium and plated at medium (C, D) or high (E, F) densities respectively. The cells plated at low density, however, had attenuation in neuronal differentiation at the detected time point (G; 9 dpp). Scale: 40 µM.

The neural differentiation following the said strategy was authenticated by immunocytochemical characterizations. Early neuroepithelial stem cell markers; nestin and Sox2 showed an overlapping expression pattern in these cells monitored at various time points during differentiation (Fig. 3A–C). While, extensive expression of NCAM and A2B5, the neuronal and glial precursor markers respectively were detected as early as 4–5 dpp (Figs. S2A–F), the nestin^+^ neural rosette like structures were quite prominent within one week of differentiation (Fig. 5D). The Map2 positivity indicative of neuronal differentiation was detected at the earliest by 5–6 dpp (Fig. 6A, B). This was more pronounced by second week of plating with well extended Map2^+^ neuronal processes (Fig. 3D; Fig. 5E) and synaptophysin positivity (Fig. 3J) implicating their maturation status. In the nes-EGFP clone, the Map2^+^ and EGFP^+^ cells were present in an interspersed manner with noted concurrence in their generation at the detected periods, thus indicating indirectly to an asynchrony in the neural differentiation pattern (Fig. 5E). This 2D differentiation strategy could also support the generation of functionally defined 5-HT^+^ serotonergic and TH^+^ dopaminergic neurons (Fig. 3E, F) and GFAP^+^ astrocytes (Fig. 3G) with a minor population of O4^+^ oligodendrocytes (Fig. 3H) without any exogenous inducers. In fact, the gliogenesis was detected at a latter stage (9 dpp or later). However, the conditions seemed sub-optimal for oligodendroglial differentiation suggesting that oligodendroglia might require exogenous supplementation of factors.

**Figure 7:**
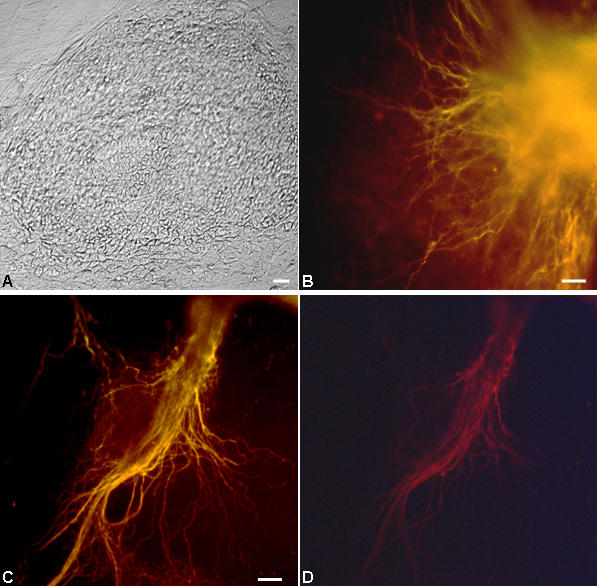
The differentiation of human ES cells into neurons. An undifferentiated human ES cell colony grown on mitotically inactivated mouse embryonic fibroblast monolayer (A). The human ES cells were differentiated into neurons (d25) in the KR medium showing Map2 positive staining (B). Some of the Map2^+^ neurons (C) were also TH^+^ (D) indicating their dopaminergic neuronal subtype status. Scale: 30 µM.

The human ES cells (Fig. 7A) exhibited neuronal differentiation at 2–3 weeks post-plating also in KR medium as ascertained by Map2 expression (Fig. 7B). Interestingly, some of these MAP2^+^ neurons were also TH^+^ indicating their dopaminergic neuronal status (Fig. 7C, D).

The immunocytochemical characterization was also carried out on cells plated at varying densities to validate our qualitative microscopic observation and ascertain whether cell density would have pronounced effect, if any, on neural differentiation. The immuno-stained pattern did corroborate well with our qualitative microscopic observation validating the cell density dependent neural differentiation (Fig. 6A–G). The neuronal differentiation with detectable Map2 positivity was observed as early as 5–6 dpp in both medium and high density plated cells (Fig. 6A, B) that became quite extensive by 9 dpp (Fig. 6C–F). However, it remained a rare phenomenon in cells plated at 2 K density even at latter stages of differentiation with or without RA (Fig. 6: compare G with C–F).

### Influence of Growth factors on neural differentiation

The growth factors through the activation of their cognate signaling cascades influence various developmental events. We used the medium density culture that was found optimal for neural differentiation to delineate whether the extrinsic influence of growth factors would further evoke neurogenic efficiency of naive ES cells during differentiation. While, bFGF promoted relatively higher (5–10% compared to control) Map2^+^ neurons generation, the GFAP^+^ astrocytes were comparatively higher (∼8–10% compared to control) with CNTF treatment. However, the overall neurogenesis remained comparable (Fig. S3) among the control and the treated groups thus suggesting that, the ES cells differentiating into neural lineage following the said strategy could generate optimum neural derivatives without any exogenous instructive factors.

### Molecular Determinants

**Figure 8:**
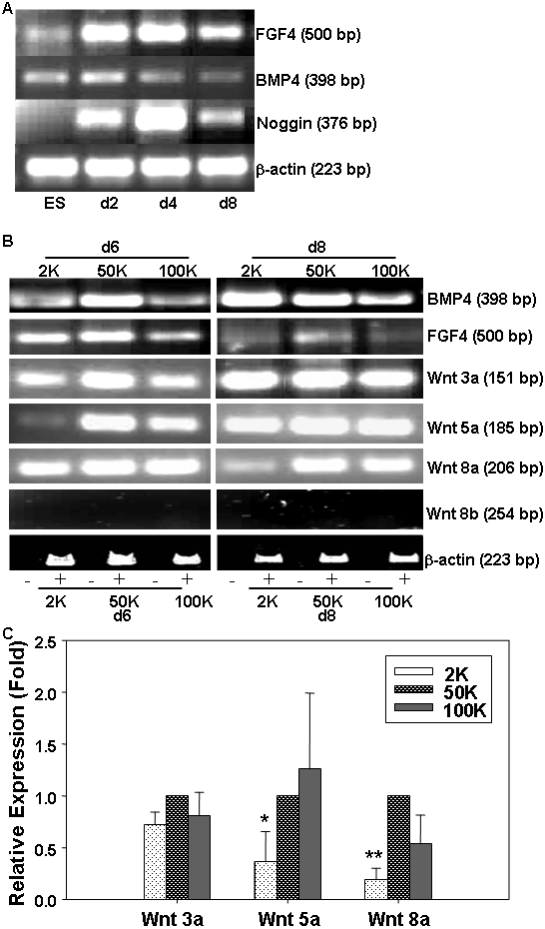
The RT-PCR analysis for secretory factors during neurogenesis in cells plated at varying densities. (A): BMP4, FGF4, Noggin expression status in undifferentiated ES cells and during various days of differentiation in cells plated at medium (50 K) density. β-actin gene served as a housekeeping positive control. (B): The expression status of BMP4, FGF4, Wnt3a, Wnt5a, Wnt8a, Wnt8b at d6 and d8 of differentiation in cells plated at varying densities. The bottom row shows the housekeeping β-actin control with (+) and without (−) reverse transcriptase in the reactions. (C): The semi-quantitative (densitometry: Wnt 3a) and quantitative (Wnt 5a, Wnt 8a) detection of Wnt transcripts relative to β-actin, keeping the relative expression count at 50 K density as 1. Data are represented as mean+/−SEM (n = 4–5) with P<0.05 (Wnt5a) and <0.01 (Wnt8a) using paired t- test for comparing the transcripts between 2 K and 50 K groups.

The variation in the density dependent neural maturation from naïve ES cells brought forth to an interesting paradigm implicating the influence of certain intrinsic secretory regulatory molecules on neural commitment and subsequent differentiation. Accordingly, we investigated the expression status of some of the key players such as BMP4, FGF4, noggin and Wnt to assess their contributory role in modulating neurogenesis. The RT-PCR was carried out on RNA samples isolated at different time periods both prior to and during differentiation from cells plated at varying densities. As seen in Fig. 8, we could detect FGF4 and BMP4 at 4, 6 and 8 dpp during differentiation and the same at low levels in ESCs. Noggin was detected only during differentiation and not in undifferentiated ESCs, while Wnt 8b was not detected in any (Fig. 8A, B). The Wnt3a, Wnt5a and Wnt8a transcripts were detected at both d6 and d8 of differentiation (Fig. 8B). However, the expression was relatively low in 2 K plating density cells at early time point (6 dpp) compared to the higher density plated ones that was ascertained by real-time (Wnt 5a and 8a) and semi-quantitative (Wnt 3a) RT-PCR (Fig. 8C). Interestingly, 6 dpp was the time when the Map2^+^ neurons started appearing at higher densities but not in 2 K plated cells thus implying a probable modulatory role of Wnt on density dependent neuronal differentiation.

### Quantitating Neurogenesis

**Figure 9:**
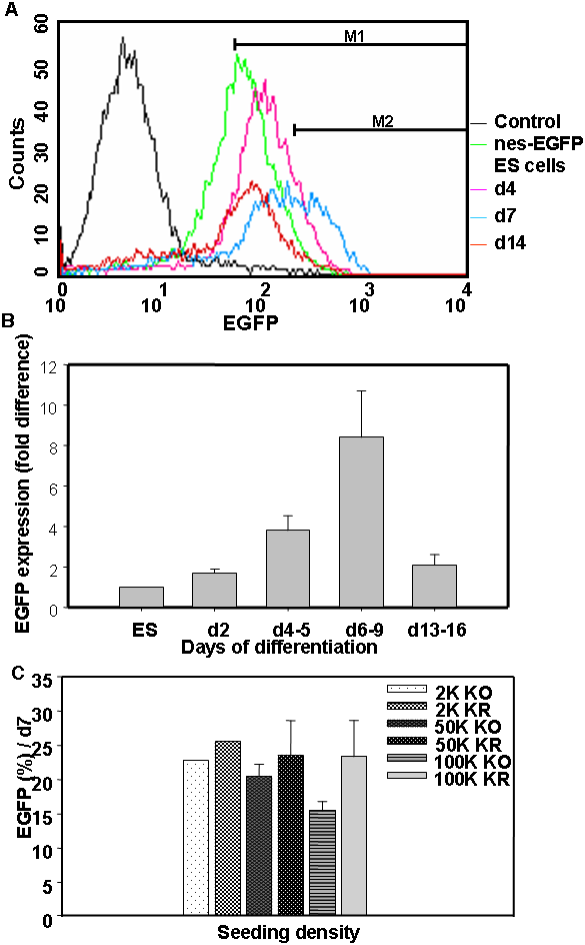
The quantification of EGFP^+^ neural progenitors by flowcytometry. (A): The nes-EGFP cells cultured in monolayer generated bright EGFP^+^ neural progenitors (M2 population) with maximum number being detected at d7. The M1 population included also the weak EGFP^+^ cells representing the differentiating population. (B): The bar diagram shows the neural progenitor generation profile comparing the EGFP population at the M2 window at different time points during differentiation keeping the ES cells value as 1 (n = 4–6). The EGFP^+^ cells peaked during 6–9 dpp, the optimum time window for efficient neural progenitor generation during differentiation followed by a decrease in the second week. (C): The bar diagram shows the influence of plating density and RA exposure of cells on EGFP^+^ neural progenitor generation at 7 dpp. Data are represented as mean+/−SEM (n = 4–7).

Further, complete neurogenesis was studied in a stepwise manner in the monoculture system to understand the probable time window for neuronal progenitor generation and subsequent maturation. Hence, we used the nes-EGFP clone and quantified the EGFP^+^ neural progenitors by flowcytometry. Our data revealed an initial increase in the EGFP expression attaining the peak during 6–9 dpp followed by its down-regulation on subsequent days (Fig. 9A–C). A distinct population (M2: 20+/−1.7%) of developing neural progenitors was seen by one week of differentiation with 6–10 fold higher EGFP intensity compared to the basal level detected in ES cells (Fig. 9A, B). Subsequently, there was drastic decrease in this M2 population (4.05+/−1.6%) in the second week of plating (Fig. 9B) indicating the maturation of differentiating neurons and glia that retained either very weak or no EGFP; a pattern that corresponded well with the endogenous nestin expression. Thus, the M2 population emphasized a time window of one week for optimum neural progenitor generation in the described monoculture strategy. Interestingly, this window was similar to our earlier finding on EB based neural differentiation [17].

With the rationale that the density would influence neurogenesis by dilution/concentration of some of the auto-/paracrine effectors, the nes-EGFP cells were plated at varying densities (2 K, 50 K and 100 K) on the gelatin coated culture dishes. FACS analysis was also carried out on these cells to determine the density dependency on neural progenitor generation and maturation. The number of EGFP expressing neural progenitors was comparable (M2: 20–23%) at one week irrespective of the plating densities except for 100 K (15+/−1.361%) indicating that the seeding density might have a minor influence on neural commitment (Fig. 9C). Certain degree of cell death seen with high density cultures might have influenced the lower percentage in the progenitor population in 100 K plated cells. The number of EGFP^+^ cells remained comparatively higher (23–30%) with RA exposure at the respective plating densities (Fig. 9C). Moreover, the effective time window of 6–9 dpp for optimal neural progenitor generation also remained same irrespective of the plating densities tested (unpublished data).

Further to monitor the differentiation profile, concurrent investigations were carried out either by the flowcytometry quantification or by selecting 5–10 random fields and counting various neural populations relative to the DAPI positive total number of cells in the medium and high density plated cultures. While, 25–30% of mature neurons were detected by first week (9 dpp), it ranged from 70–80% or more (76.8+/−6.9%) on second week. Similarly, the GFAP population also showed an increase from 3–5% to ∼20% by second week of differentiation. Contrary to this, there was a decline in the A2B5 population from first week (∼64%) to second (8–10%). Our differentiation strategy also yielded ∼25% dopaminergic neurons (TH^+^). A comparable number of Map2^+^ cells were detected in both densities with marginal increase (5–10%) in KR medium at one week time period that became almost identical (∼80%) by two weeks post-plating with or without RA. Taken together, the medium density was found ideal for both progenitor generation and neural maturation associated with extensive neurite processes thus suggesting a vital influence of initial seeding density on neurogenic efficacy and the medium being serum free could still support this.

### Intervening neural stem/progenitor stage and stemness

**Figure 10:**
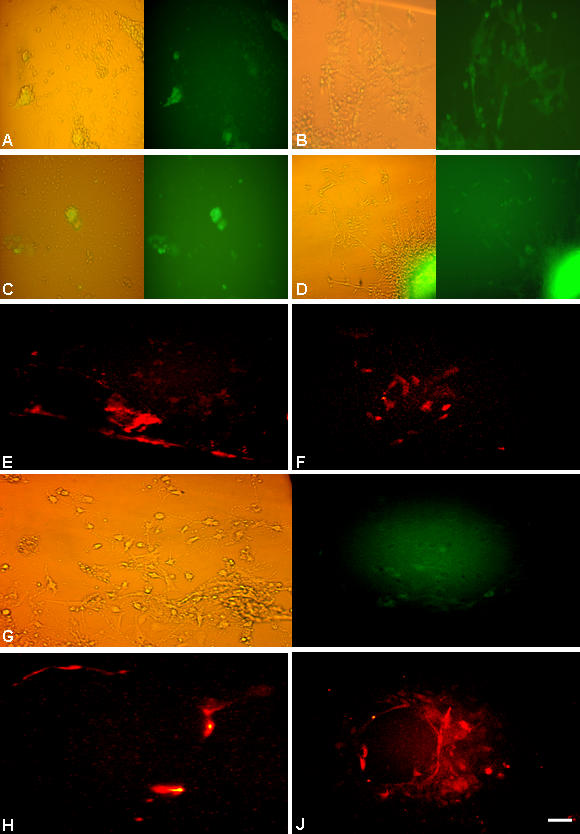
The differentiation profile of neural progenitors purified by MACS. The nes-EGFP cells 8 dpp were subjected to MACS, the A2B5^+^ eluent (A–D) and SSEA1^−^ flow through (G) were collected and grown in culture. The A2B5^+^ cells cultured in either DM (A: 6 dpp; B: 10 dpp) or KO medium (C: 6 dpp; D: 10 dpp) exhibited proliferation and differentiation with better differentiation in KO (D) that showed both Map2^+^ neurons (E) and GFAP^+^ astrocytes (F). Similarly the SSEA1^−^ cells when cultured in KO medium (G) underwent differentiation into Map2^+^ neurons (H) and GFAP^+^ astrocytes (J). Scale: 30 µM.

To ascertain the stage at which the unspecified ES cells acquire the neural fate, we stained the cells with neural stem cell/progenitor markers such as Sox1, Sox2 and nestin both prior to and after inducing differentiation. Sox1, a specific marker for neural committed population [19] was detected co-localized with nestin at 2 dpp with optimum expression seen around 4 dpp. Subsequently, it showed a downward trend with undetectable level by d7 and after (Fig. 5A–C, F). However, both nestin and Sox2 were detected in ES cells itself although at a low level [17; unpublished data]. Their expression was more pronounced from d2 onwards and became less at the end of second week corresponding with the quantitative EGFP profile (Fig. 3A–C; Fig. 9B). Thus, together with the quantitative flowcytometry observation we could discern that, the neural commitment following the said strategy took place as early as 2 dpp, while the optimal time window for neural progenitors resided during 6–9 dpp. Accordingly, the cells at d3/8 were dispersed and re-plated with KO or DM to assess the multi-potentiality and stemness of neural progenitors [4], [19], [20]. Interestingly, the re-plated cells also exhibited proliferation and neural differentiation (Fig. S3G) supporting the existence of intervening neural stem cell stage during ES cells' differentiation [4], even though a minor population of coexisting undifferentiated ES cells in the culture could not be ruled out at this stage. This led us further to isolate these intervening cells from differentiating ES cells by MACS using A2B5, NCAM and SSEA1 antibodies and with positive and negative selections respectively. The purification of NCAM^+^ population was sub-optimal in our hand. However, the A2B5^+^ (Fig. 10A–D) and SSEA1^−^ (Fig. 10G) cells upon plating on DM or KO medium exhibited both proliferation and differentiation. The cells isolated by negative selection using SSEA1 antibody differentiated into both neurons and astroglia (Fig. 10H, J). Even the A2B5^+^ cells that are supposed to be the glial progenitors also generated both neurons and astroglia upon plating (Fig. 10E, F).

## Discussion

The ES cells in undifferentiated state are regarded as naïve. These cells attain commitment to specific cell fates upon appropriate stimuli conferred by both cell intrinsic and extrinsic means, that *in vitro* is triggered mostly by cell–cell interaction. Hence, the naïve ES cells are allowed to aggregate, a process that results in three dimensional structures called EBs, to initiate cellular communication and differentiation [17], [21]. Following the same rationale we tried to bring about cellular net working in a 2D set up allowing the ES cells to proliferate and form colonies in a medium containing FBS but without LIF. Interestingly, this 2D cellular interaction through priming with FBS followed by culturing in KO – the medium that is devoid of any growth factors and neurogenic inducers could facilitate gaining requisite signals in promoting neural commitment as ascertained by Sox1 positivity. The attainment of neural fate thus underscored the involvement of LIF in the undifferentiated ES cell maintenance, the absence of which led to the differentiation [21] and in parallel signified the fact that, the neural differentiation from ES cells might be a preferred event.

A number of reports [15], [17], [22]–[24] have indicated the ES cells' differentiation into neurons by culturing those on stromal cell monolayer or with neurogenic inducers (RA, bFGF, BMP inhibitors) in the medium. However, it might be ideal to have a basic platform developed without any exogenous influence to assess without ambiguity the causal players during development. Only in a recent study [25] the ES cells were shown to attain a neural identity when cultured in PBS. However, the cell attachment and survival was a major impediment there. Here we have demonstrated the neural differentiation from ES cells in a medium devoid of these inducers emphasizing only to the intrinsic properties of these cells underlying this process. The differentiation was induced by LIF withdrawal and in low serum conditions and that was sufficient to induce the expression of the neuroepithelial stem cell markers. With the hypothesis that, the withdrawal of growth factors normally found in serum could be sufficient for neurogenesis to be induced, we allowed the dissociated ES cells to have an exposure to serum conditions initially and then substituted that with serum replacement medium. The latter medium is thought to be devoid of any growth factors and retinoid derivatives which are known to induce neurogenesis. The priming of cells for one day to low serum also had comparable response. Similarly, culturing the ES cells in KO medium from d0 also resulted in extensive neural differentiation in spite of a significantly low number of adherent colonies remaining in the dish (unpublished data). Thus, it becomes imperative to presume that the priming with low serum might be a dispensable event. The serum might have helped primarily in better cell adhesion to the dish and thereby promoting subsequent neural differentiation in KO medium. Thus, not only withdrawal of LIF but the exposure of cells to KO medium seemed essential and could be used in combination to accomplish efficient neural differentiation from ES cells without exogenous factors. This strategy might be one of the simplest ones for obtaining enriched population of neural progenitors and differentiated neurons from ES cells that would further help gaining insight into cues and factors involved during neural differentiation. In fact, it would be realistic to narrow down the components present in the serum replacement formulation to assess the influence of specific factors on neurogenesis and also to try several ECM components for facilitating improved cell adhesion, though laminin/fibronectin substrates seemed to promote non-neural cells compared to gelatin [3].

The ES cell derived neurogenesis would include both the committed neural stem cells/progenitors and the differentiated progenies. This demanded the requirement of properly demarcating and quantifying the neural progenitor population from the differentiating neurons using nestin, the neuroepithelial stem cell marker [26]–[29] to understand the influence of conditions on both the events independently leading to ultimate maturation. Other than the rationale of avoiding known external factors in the medium we also deduced the neighbouring effect of these cells on each other in terms of their density to influence the neurogenesis, focusing directly on their intrinsic characteristics. While plating density had no appreciable influence on the EGFP^+^ neural progenitor generation, we could decipher an interesting synergy between the seeding density and the neuronal maturation with the differentiation impairment in low density culture and high density ones showing higher cell death. Interestingly, the medium density culture that we found optimal for neurogenesis was also similar to the reported [3] density (0.5×10^4^/cm^2^). The results from the influence of density on the differentiating cells thus directly portrayed that, the intrinsic factors were probably secreted and regulated the differentiation process in an auto-/paracrine fashion. The lack of appreciable influence of exogenous growth factors on neural differentiation indeed substantiated this further.

A number of factors have in fact been implicated in modulating neurogenesis [29]–[36]. Interestingly, the FGF signal requirement for neural induction starts even prior to gastrulation [32]. A preliminary assessment of probable modulators has revealed a correlation of plating density on FGF4 transcripts expression while with respect to noggin no clear pattern could be deduced (unpublished data). It might be likely that, the FGFs bear the master switch and the threshold of these would tilt the neural differentiation efficiency. Similarly, the Wnt transcripts were low at 2 K plating density at a stage (d6) when with other densities the Map2^+^ neurons were first detected. Considering the density dependent positive influence of Wnt on neuronal differentiation [33], we deciphered the lack of Wnt threshold might be adversely affecting the neuronal differentiation in low density culture. The same might have held true in serially diluted single cell culture which also lacked neuronal differentiation. A number of reports also indicate the role of BMPs during neurogenesis. It is known that, in xenopus the cell dissociation of the presumptive ectoderm dilutes BMP signals and thus directs the neuronal differentiation rather than the epidermal one [13], [37], [38]. The BMPs also exert concentration dependent inductive influence on dorsal neural cell types [34]. Moreover, BMP on one hand helps in the maintenance of ES cells [3], [39] while on the other promotes gliogenesis during neural differentiation [19]. Our observation on BMP4 transcripts detection in ES cells and at different days during neural differentiation indicated that, the endogenous BMP was non-interfering during neurogenesis [3]. Moreover, other than these secretory factors the plating density's influence on differentiation might also have been triggered by the neighbouring cells' crosstalk through Notch and hedgehog signaling [40]–[45]. Further work would be needed to verify this.

RA is one of the key players that exerts pleiotropic influence during development [34]–[36] and promotes neuronal differentiation from ES cells through enhancement of beta-catenin signaling [19]. In the present investigation, we showed that, RA not only expedited the neurogenic progression, its supplementation in the medium promoted the generation of neural progenitors and the differentiated neurons [17]. In fact, RA might overcome the neural inhibitory signals and direct the cells to neural lineage since exposure to RA even in presence of serum could guide the ES cells to structure neural differentiation. This effect directly portrays that; the endogenous players in concert with RA might be exerting their influence on promoting neurogenesis. It would indeed be interesting to identify the RA downstream factors that strongly direct the cells for neural specification and differentiation, although we have succeeded in achieving efficient neurogenesis without RA supplementation. Possibly, a threshold of such factors including FGFs, Wnts, RA and their synergistic effect promote optimum neurogenesis. Thus, it would be imperative to assess whether the activation/inhibition of these signals would suffice to induce neurogenesis in cells or these would only help the already induced neural cells to form neurons.

Conti et al, [19] have reported a clonogenic neural stem cell population derived from ES cells. In line with theirs we could also detect specific time windows for Sox1^+^ neural committed population that resided around 4 days of initiation of differentiation while the effective time window for nestin^+^ neural progenitors was about 6–9 dpp suggesting the existence of developmental hierarchy during early neurogenesis. In fact, Sox1^+^ stage cells could be split and differentiated upon re-plating without affecting their survival (unpublished data). Earlier [17], we have also shown in real time the self-renewing neural progenitors, differentiating neurons and astrocytes generated from EBs dispersed during the one week time window. This prompted us to purify the progenitors using MACS and differentiate those successfully into neurons and astrocytes thus demonstrating further the efficacy of the devised monoculture strategy in stage specific cell type(s) isolation and their characterization. Moreover, our observation also envisaged that common progenitors might exist for neuro- and gliogenesis.

In fact, the neural induction has remained a debatable issue pertaining to whether it is a mere default process or it involves precise instructive cues. The present investigation has highlighted a combinatorial cell intrinsic and neighboring effect that influenced the cells to undergo neural specification and differentiation from ES cells. While the neural commitment was density independent, the maturation efficiency was dictated by the initial cell plating density thus questioning how appropriate could it be to regard neurogenesis that would include both commitment and maturation; as a default transition in the absence of inhibitory signals [13], [46]? It might be likely that, the whole machinery of neural induction remains in a proactive state and the withdrawal or the absence of the influence of some factors might be sufficient for this machinery to be active. Conversely, the density dependent neural differentiation indicated it to be more likely of a neighbouring effect and hence instructive, that influenced the cells to acquire particular phenotype. Indeed this brings forth to an interesting paradox. In case the default hypothesis is true, it should then be limited to the commitment stage, but not involving maturation. Interestingly, the ES cells do express the transcripts of other germ layers [25]. In this context, providing both permissive and instructive cues supporting other lineages might also help directing the ES cells *in vitro* to different lineages other than the neural and the conditions opted till date might not be ideal for the same. Never-the-less, incase the counter view on FGF mediated neural specification [3] is true, then possibly autocrine and/or paracrine stimulation/inhibition might be the critical determinants underlying this process. For the maturation, however, cellular crosstalk at the initiation stage would be mandatory. Taken together, it could very well be inferred that while neural commitment might be a default state or with autocrine stimulus being sufficient for the same; the maturation efficiency would depend on the cellular communication and community effect commenced and exerted from the commitment/specification stage itself. Thus, our investigation has shed some light on this long standing issue on neurogenesis, putting forth an interesting amalgamation of both permissive and instructive cues to underlie the overall process of neurogenesis.

## Materials and Methods

### ES cells maintenance and differentiation

The murine ES cells, line D3 (ATCC) were maintained routinely on STO fibroblasts and cultured without fibroblast prior to differentiation as described [47]. We have established a fresh batch of nes-EGFP stable ES cell clones by introducing the h-nestin EGFP construct [human nestin intron II (a kind gift from Dr. Lendahl) sub-cloned into pEGFP1 vector] to the D3 ES cells by electroporation following the protocol [17] and characterized those. For monoculture set up, the enzymatically dispersed single cells were seeded on gelatin coated dishes using DMEM with 10% FBS and other additives, except LIF (DM). The medium was substituted with Knockout-DMEM (KO) on d2 that contained all the additives and Knockout serum replacement (Invitrogen) in place of FBS and was supplemented with (KR) or without 100 nM all-trans retinoic acid (RA). Subsequently, the cells were maintained in KO with medium replenishment carried out on every other day or depending on the cellular demand. In parallel, the cells were also maintained in DM or in ITSFn [48] medium either with or without RA to assess the optimum culture requisites for 2D culture. For comparing this with the 3D culture, the EBs were generated in hanging drop for 2 days using DM followed by the suspension culture with 100 nM RA for 3–5 days and plating on d5/7 following the protocol [47]. On 2 dpp, the DM was replaced with KO and the cells were continued culturing on the same.

Following similar strategy of monoculture, the in-house established and partially characterized human ES cell line/outgrowth, RH1; derived from a discarded abnormal embryo with three pronuclei [with institutional ethical committees' (IEC), National Centre for Cell Science approval and informed parental consent (oral; since the embryo was unsuitable for implantation and was meant to be discarded] was also differentiated into neural lineage. The difference being, the RH1 ES cells instead of single dispersed cells were grown in clumps for differentiation in medium containing 20% FBS and that was substituted with KR medium on day 2–3 after plating.

### Plating density and neural differentiation

To discern the influence of cell density on neural differentiation, the D3 ES cells and one of the representative nes-EGFP clones (17) were plated at varying densities; [low: 1–2×10^3^cells (1–2 K), medium: 5–6.5×10^4^ cells (50–65 K), and high: 1–1.3×10^5^ cells (100–130 K)] on 35 mm dishes (9.8 cm^2^) and were monitored for their qualitative and quantitative differences. The growth characteristics and the differentiation were monitored under the microscope (Nikon, TE2000U) and were characterized. Various growth factors were added on d2 to the medium density culture in KR medium at the described concentrations (see figure legend), to assess their influence on neural differentiation. The RNA was isolated from cells during different stages of differentiation using Trizol reagent (Invitrogen) and was subjected to reverse transcription PCR (RT-PCR) [17] to assess the factors influencing neurogenesis (The primer sequences could be made available upon request). For quantification, either semi-quantitative RT-PCR followed by densitometry analysis or quantitative real time PCR using SYBR Green reagent (BioRad) was performed. Relative expression levels were calculated normalizing to β-actin transcription. Statistical analyses were carried out using paired t-test.

### Immunophenotyping

The cellular phenotypes attained during ES cells differentiation into neural lineage in a monolayer culture were discerned by immunocytochemistry using antibodies against neurons and glia following the standard protocol [47]. The cells were exposed to either of the primary antibodies; anti-nestin (Rat-401, DSHB, University of Iowa), anti-MAP2, anti-TuJ1, anti-synaptophysin, anti-tyrosine hydroxylase (TH), anti-GFAP (all from Sigma), anti-Sox1, anti-Sox2, anti-NCAM, anti-A2B5, anti-5HT or anti-O4 (all from Chemicon International) and Cy3- or Cy5- conjugated secondary antibodies for fluorescent labeling. In each case the negative control was performed with the substitution of respective primary antibodies with FBS or pre-immune goat serum. The slides were observed under a laser scanning confocal microscope (LSM 510, CarlZeiss) to detect EGFP as well as Cy3-/Cy5-labeling. Some of these the immuno-stained cells at various time points during differentiation were also subjected to flowcytometry using FACSCalibre and CellQuestPro software (Becton Dickinson) to quantitate various neural populations. In parallel, about 5–10 random fields each were considered for quantifying the Map2^+^/GFAP^+^differentiated neuronal population among the Dapi^+^ cells during ES cells differentiation from a minimum of three experiments.

### Flowcytometric Quantification

The EGFP expression in nes-EGFP clones was analyzed at various developmental stages using a FACSVantage flowcytometer (Becton Dickinson) equipped with a 488 nm argon-ion-laser (150 mW) as described [17]. About 10,000 viable cells were analyzed per sample following isolation while untransfected D3 ES cells served as the negative control. The emitted fluorescence of EGFP was measured in log scale at 530 nm (FITC band pass filter) and the analyses were performed using CellQuestPro software (Becton Dickinson).

### Self Renewal, Cell Dissociation and re-plating

To assess whether the ES cells would involve an intervening neural stem cell stage that would have self-renewing ability, we dispersed the differentiating ES cells and re-plated those on gelatin coated dishes. The dispersion was carried out either enzymatically using Trypsin-EDTA or mechanically during early time points (3 dpp and 8 dpp). While the mechanical isolation yielded cells in clumps, the enzymatic dispersion gave single cells. Subsequently, the cells were cultured in either DM or KO medium.

### Isolation and purification of neural progenitors

To purify the neural progenitors, the plated cells at d6-8 during differentiation were enzymatically dispersed and were allowed to revive their membrane integrity by incubating those in DM for 2 hours in the CO2 incubator. The cells were washed subsequently with PBS and subjected to MACS to purify the neural progenitors following the specified protocol (Miltenyi Biotec). In brief, the cells were suspended in 500 l of pre-chilled MACS buffer (PBS, pH. 7.2 with 0.5% BSA and 2 mM EDTA) and were incubated with either of the following primary antibodies (A2B5, NCAM, SSEA1) for 30 minutes at 4°C. The unbound antibodies were removed by washing thoroughly and the cells were suspended in 80 l MACS buffer. The cell suspension was added with magnetic beads conjugated secondary antibody slurry (20 l) and was incubated for further 20 minutes at 4°C. The washing step was repeated and finally the cells were suspended in 500 l of MACS buffer followed by loading onto a separation column placed under the magnetic field that was pre-equilibrated with MACS buffer. The flow-through was collected (−ve fraction) and the column was removed from the magnetic field after washing with the MACS buffer. Subsequently, the bound cells (+ve fraction) were eluted with MACS buffer (1 ml) using the plunger. The cells from both the +ve and the –ve fractions were pellet down and the cell pellets were suspended in KO medium. The cells were further maintained in DM or KO medium to assess their growth and differentiation characteristics.

## Supporting Information

Figure S1 The neural differentiation in ES cells depended on the initial plating density of cells. The extent of cell growth seen in nes-EGFP cells at d2 with 2 K (A), 60 k (B) and 130 K (C) plating densities. The neural differentiation in cells at 2 K plating density was observed occasionally in KO (D) or KR (E) medium at second week post-plating (d2+10) and remained confined to cell dense areas only. However, cells (D3 ES) plated at higher density (60 K) exhibited extensive neural differentiation with well sprouted neural processes by second week (F–H). Single cells migrating away from the cell cluster also retained neurogenic differentiation potential in those (G, H). Scale: 30 µM
(0.95 MB TIF)

Figure S2 The ES cells during differentiation showed the neuronal and neuro-glial progenitor markers, NCAM and A2B5 respectively. The EGFP^+^ neural progenitors showed surface expression (7 dpp) of NCAM (A,B) and A2B5 (C,D) when cultured in KO or KR medium respectively. Interestingly, both these markers were expressed as early as 5 dpp as seen in D3 ES cells (E: NCAM; F: A2B5). Scale: 40 µM.
(0.77 MB TIF)

Figure S3 Exogenous supplementation of growth factors into the medium did not have pronounced effect on neural differentiation from ES cells *in vitro*. The D3 ES cells cultured in KR medium without (A) or with bFGF (10 ng/ml) (B), NGF (10 ng/ml) (C), BDNF (10 ng/ml) (D), CNTF (2 ng/ml) (E) and FGF8 (100 ng/ml) (F) respectively showed similar neural differentiation pattern at 2 weeks (14 dpp). The inset represents the TuJ1^+^ neurons in each. (G): The ES cells cultured in KR medium when dispersed on d3 and re-plated on DM followed by changing to KO on d2 of re-plating displayed extensive neural differentiation with well sprouted processes as seen on d11 of re-plating. Scale: 30 µM.
(0.91 MB TIF)

## References

[1] Hemmati-Brivanlou A, Melton D (1997). Vertebrate embryonic cells will become nerve cells unless told otherwise.. Cell.

[2] Munoz-Sanjuan I, Hemmati-Brivanlou A (2002). Neural induction, the default model and embryonic stem cells.. Nat Rev Neurosci.

[3] Ying QL, Stavridis M, Griffiths D, Li M, Smith A (2003a). Conversion of embryonic stem cells into neuroectodermal precursors in adherent monoculture.. Nat Biotechnol.

[4] Tropepe V, Hitoshi S, Sirard C, Mak TW, Rossant J (2001). Direct neural fate specification from embryonic stem cells: a primitive mammalian neural stem cell stage acquired through a default mechanism.. Neuron.

[5] Niwa H, Burdon T, Chambers I, Smith A (1998). Self-renewal of pluripotent embryonic stem cells is mediated via activation of STAT3.. Genes Dev.

[6] Matsuda T, Nakamura T, Nakao K, Arai T, Katsuki M (1999). STAT3 activation is sufficient to maintain an undifferentiated state of mouse embryonic stem cells.. EMBO J.

[7] Ying QL, Nichols J, Chambers I, Smith A (2003b). BMP induction of Id proteins suppresses differentiation and sustains embryonic stem cell self-renewal in collaboration with STAT3.. Cell.

[8] Thomson JA, Itskovitz-Eldor J, Shapiro SS, Waknitz MA, Swiergiel JJ (1998). Embryonic stem cell lines derived from human blastocysts.. Science.

[9] Daheron L, Opitz SL, Zaehres H, Lensch WM, Andrews PW (2004). LIF/STAT3 Signaling Fails to Maintain Self-Renewal of Human Embryonic Stem Cells.. Stem Cells.

[10] Chambers I, Colby D, Robertson M, Nichols J, Lee S (2003). Functional expression cloning of Nanog, a pluripotency sustaining factor in embryonic stem cells.. Cell.

[11] Mitsui K, Tokuzawa Y, Itoh H, Segawa K, Murakami M (2003). The homeoprotein Nanog is required for maintenance of pluripotency in mouse epiblast and ES cells.. Cell.

[12] Suzuki A, Raya A, Kawakami Y, Morita M, Matsui T (2006). Nanog binds to Smad1 and blocks bone morphogenetic protein-induced differentiation of embryonic stem cells.. Proc Natl Acad Sci U S A.

[13] Streit A, Stern CD (1999). Neural induction. A bird's eye view.. Trends Genet.

[14] Bainter JJ, Boos A, Kroll KL (2001). Neural induction takes a transcriptional twist.. Dev Dyn.

[15] Okabe S, Forsberg-Nilsson K, Spiro AC, Segal M, McKay RD (1996). Development of neuronal precursor cells and functional postmitotic neurons from embryonic stem cells in vitro.. Mech Dev.

[16] Lee SH, Lumelsky N, Studer L, Auerbach JM, McKay RD (2000). Efficient generation of midbrain and hindbrain neurons from mouse embryonic stem cells.. Nat Biotechnol.

[17] Lenka N, Lu ZJ, Sasse P, Hescheler J, Fleischmann BK (2002). Quantitation and functional characterization of neural cells derived from ES cells using nestin enhancer-mediated targeting in vitro.. J Cell Sci.

[18] Yamazoe H, Kobori M, Murakami Y, Yano K, Satoh M (2006). One-step induction of neurons from mouse embryonic stem cells in serum-free media containing vitamin B12 and heparin.. Cell Transplant.

[19] Conti L, Pollard SM, Gorba T, Reitano E, Toselli M (2005). Niche-independent symmetrical self-renewal of a mammalian tissue stem cell.. PLoS Biol.

[20] Clarke DL, Johansson CB, Wilbertz J, Veress B, Nilsson E (2000). Generalized potential of adult neural stem cells.. Science.

[21] Burdon T, Smith A, Savatier P (2002). Signalling, cell cycle and pluripotency in embryonic stem cells.. Trends Cell Biol.

[22] Bain G, Kitchens D, Yao M, Huettner JE, Gottlieb DI (1995). Embryonic stem cells express neuronal properties in vitro.. Dev Biol.

[23] Kawasaki H, Mizuseki K, Nishikawa S, Kaneko S, Kuwana Y (2000). Induction of midbrain dopaminergic neurons from ES cells by stromal cell-derived inducing activity.. Neuron.

[24] Kim DW, Chung S, Hwang M, Ferree A, Tsai HC (2007). Stromal cell-derived inducing activity, Nurr1, and signaling molecules synergistically induce dopaminergic neurons from mouse embryonic stem cells.. Stem Cells.

[25] Smukler SR, Runciman SB, Xu S, van der Kooy D (2006). Embryonic stem cells assume a primitive neural stem cell fate in the absence of extrinsic influences.. J Cell Biol.

[26] Lendahl U, Zimmerman LB, McKay RD (1990). CNS stem cells express a new class of intermediate filament protein.. Cell.

[27] Lothian C, Lendahl U (1997). An evolutionarily conserved region in the second intron of the human nestin gene directs gene expression to CNS progenitor cells and to early neural crest cells.. Eur J Neurosci.

[28] Lothian C, Prakash N, Lendahl U, Wahlstrom GM (1999). Identification of both general and region-specific embryonic CNS enhancer elements in the nestin promoter.. Exp Cell Res.

[29] Lamb TM, Knecht AK, Smith WC, Stachel SE, Economides AN (1993). Neural induction by the secreted polypeptide noggin.. Science.

[30] Lamb TM, Harland RM (1995). Fibroblast growth factor is a direct neural inducer, which combined with noggin generates anterior-posterior neural pattern.. Development.

[31] Linker C, Stern CD (2004). Neural induction requires BMP inhibition only as a late step, and involves signals other than FGF and Wnt antagonists.. Development.

[32] Streit A, Berliner AJ, Papanayotou C, Sirulnik A, Stern CD (2000). Initiation of neural induction by FGF signalling before gastrulation.. Nature.

[33] Otero JJ, Fu W, Kan L, Cuadra AE, Kessler JA (2004). {beta}-Catenin signaling is required for neural differentiation of embryonic stem cells.. Development.

[34] Wilson L, Maden M (2005). The mechanisms of dorsoventral patterning in the vertebrate neural tube.. Dev Biol.

[35] Irioka T, Watanabe K, Mizusawa H, Mizuseki K, Sasai Y (2005). Distinct effects of caudalizing factors on regional specification of embryonic stem cell-derived neural precursors.. Brain Res Dev Brain Res.

[36] Ribes V, Wang Z, Dolle P, Niederreither K (2006). Retinaldehyde dehydrogenase 2 (RALDH2)-mediated retinoic acid synthesis regulates early mouse embryonic forebrain development by controlling FGF and sonic hedgehog signaling.. Development.

[37] Wilson PA, Hemmati-Brivanlou A (1995). Induction of epidermis and inhibition of neural fate by Bmp-4.. Nature.

[38] Wilson PA, Hemmati-Brivanlou A (1997). Vertebrate neural induction: inducers, inhibitors, and a new synthesis.. Neuron.

[39] Qi X, Li TG, Hao J, Hu J, Wang J (2004). BMP4 supports self-renewal of embryonic stem cells by inhibiting mitogen-activated protein kinase pathways.. Proc Natl Acad Sci U S A.

[40] Artavanis-Tsakonas S, Rand MD, Lake RJ (1999). Notch signaling: Cell fate control and signal integration in development.. Science.

[41] Lai EC (2004). Notch signaling: control of cell communication and cell fate.. Development.

[42] Morrison SJ, Perez SE, Qiao Z, Verdi JM, Hicks C (2000). Transient Notch Activation Initiates an Irreversible Switch from Neurogenesis to Gliogenesis by Neural Crest Stem Cells.. Cell.

[43] Goodrich LV, Scott MP (1998). Hedgehog and patched in neural development and disease.. Neuron.

[44] Ingham PW, McMahon AP (2001). Hedgehog signaling in animal development: paradigms and principles.. Genes and Dev.

[45] Cayuso J, Ulloa F, Cox B, Briscoe J, Marti E (2006). The Sonic hedgehog pathway independently controls the patterning, proliferation and survival of neuroepithelial cells by regulating Gli activity.. Development.

[46] Moreau M, Leclerc C (2004). The choice between epidermal and neural fate: a matter of calcium.. Int J Dev Biol.

[47] Lenka N (2006). Derivation and characterization of neural cells from embryonic stem cells using nestin enhancer.. Methods Mol Biol.

[48] Andressen C, Stocker E, Klinz FJ, Lenka N, Hescheler J (2001). Nestin-specific green fluorescent protein expression in embryonic stem cell-derived neural precursor cells used for transplantation.. Stem Cells.

