# Supplementary figures and images for: Neural Induction from ES Cells Portrays Default Commitment but Instructive Maturation
Source: PLoS One. 2007 Dec 19;2(12):e1349. doi: 10.1371/journal.pone.0001349 (PMC2121127; doi:10.1371/journal.pone.0001349)

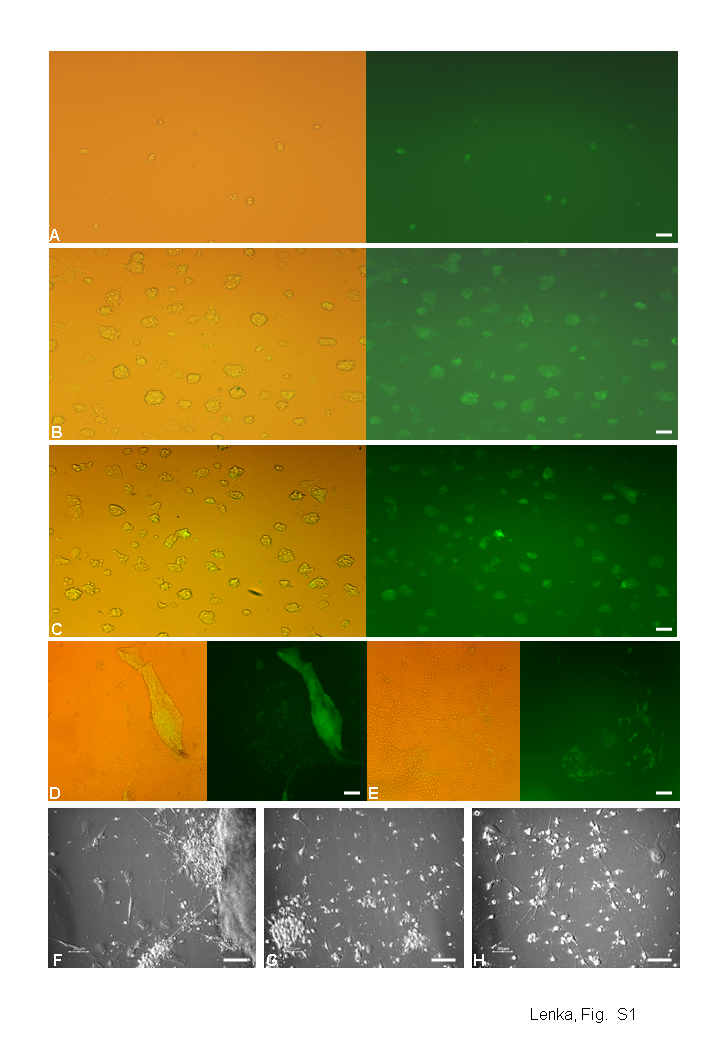

Supplement: Figure S1 — The neural differentiation in ES cells depended on the initial plating density of cells. The extent of cell growth seen in nes-EGFP cells at d2 with 2 K (A), 60 k (B) and 130 K (C) plating densities. The neural differentiation in cells at 2 K plating density was observed occasionally in KO (D) or KR (E) medium at second week post-plating (d2+10) and remained confined to cell dense areas only. However, cells (D3 ES) plated at higher density (60 K) exhibited extensive neural differentiation with well sprouted neural processes by second week (F–H). Single cells migrating away from the cell cluster also retained neurogenic differentiation potential in those (G, H). Scale: 30 µM (0.95 MB TIF) [file pone.0001349.s001.tif]

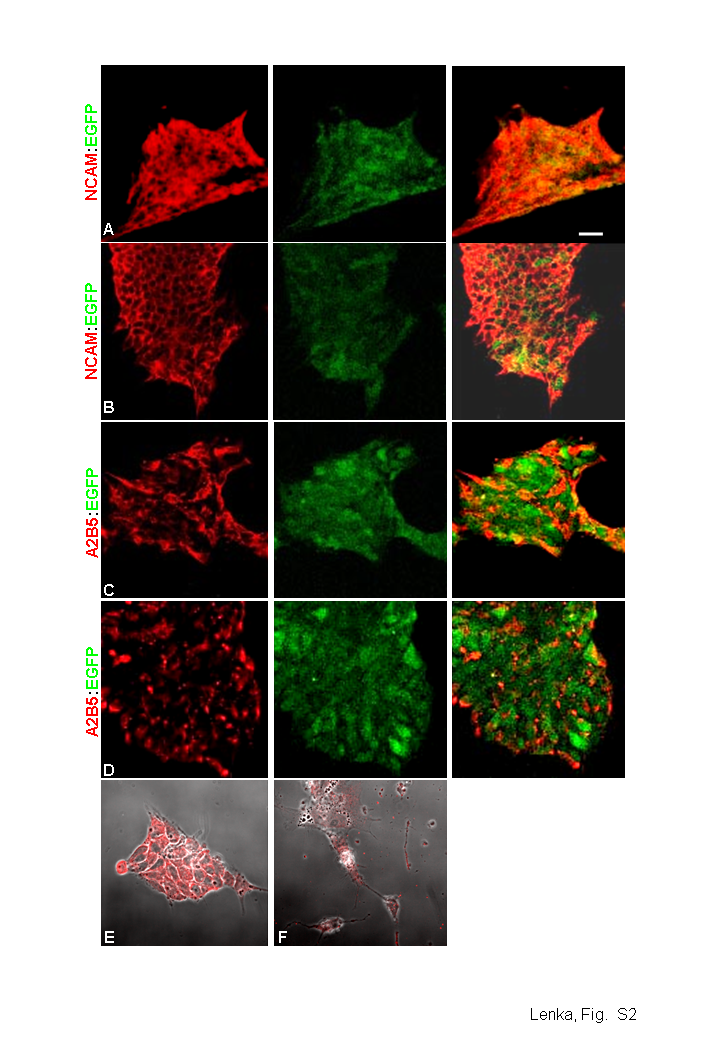

Supplement: Figure S2 — The ES cells during differentiation showed the neuronal and neuro-glial progenitor markers, NCAM and A2B5 respectively. The EGFP+ neural progenitors showed surface expression (7 dpp) of NCAM (A,B) and A2B5 (C,D) when cultured in KO or KR medium respectively. Interestingly, both these markers were expressed as early as 5 dpp as seen in D3 ES cells (E: NCAM; F: A2B5). Scale: 40 µM. (0.77 MB TIF) [file pone.0001349.s002.tif]

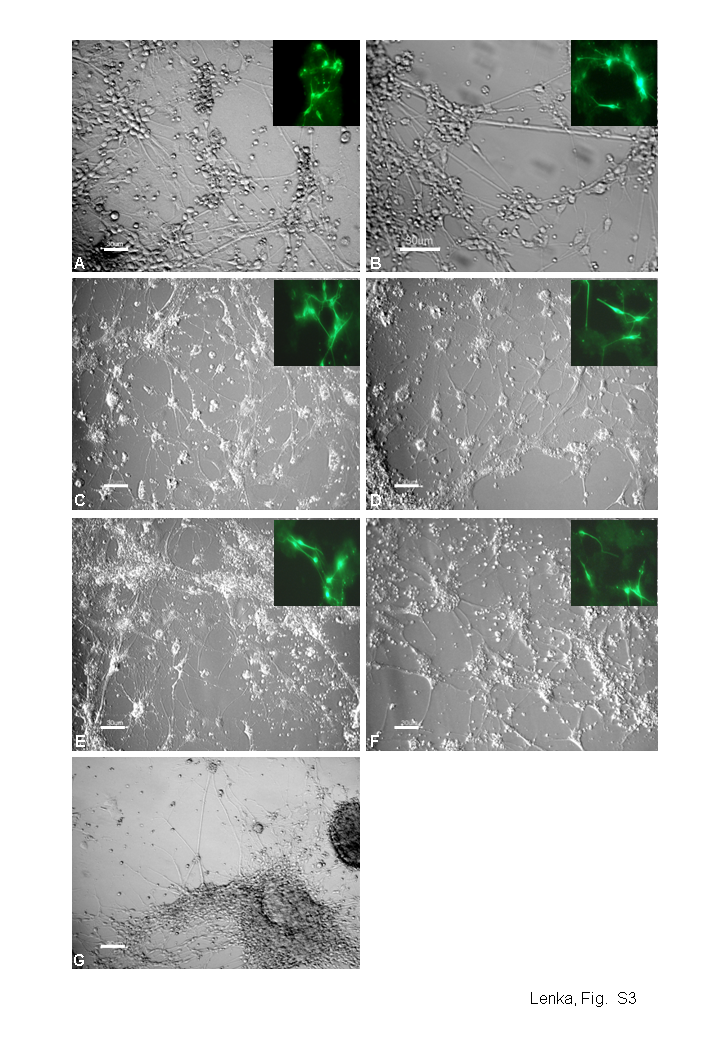

Supplement: Figure S3 — Exogenous supplementation of growth factors into the medium did not have pronounced effect on neural differentiation from ES cells in vitro. The D3 ES cells cultured in KR medium without (A) or with bFGF (10 ng/ml) (B), NGF (10 ng/ml) (C), BDNF (10 ng/ml) (D), CNTF (2 ng/ml) (E) and FGF8 (100 ng/ml) (F) respectively showed similar neural differentiation pattern at 2 weeks (14 dpp). The inset represents the TuJ1+ neurons in each. (G): The ES cells cultured in KR medium when dispersed on d3 and re-plated on DM followed by changing to KO on d2 of re-plating displayed extensive neural differentiation with well sprouted processes as seen on d11 of re-plating. Scale: 30 µM. (0.91 MB TIF) [file pone.0001349.s003.tif]
